# Supplementary material for: CDPK2A and CDPK1 form a signaling module upstream of Toxoplasma motility
Source: mBio. 2023 Aug 23;14(5):e01358-23. doi: 10.1128/mbio.01358-23 (PMC10653799; doi:10.1128/mbio.01358-23)
Supplement: Supplemental Figures — Figures S1-S6. [file mbio.01358-23-s0001.pdf]

CDPK2A and CDPK1 form a signaling module upstream of *Toxoplasma* motility

Emily Shortt[a], Caroline G. Hackett[a*], Rachel V. Stadler[b], Robyn S. Kent[b], Alice L. Herneisen[a,c], Gary E. Ward[b], Sebastian Lourido[a,c#]

[a] Whitehead Institute, Cambridge, MA

[b] Department of Microbiology and Molecular Genetics, University of Vermont Larner College of Medicine, Burlington, VT

[c] Biology Department, MIT, Cambridge, MA

* Current address: Regeneron Pharmaceuticals, Tarrytown, NY

[#] Correspondence: lourido@wi.mit.edu

SUPPLEMENTAL TABLES, FIGURES, & LEGENDS

Supplementary Table 1. List of oligos, plasmids, and strains used in this study.

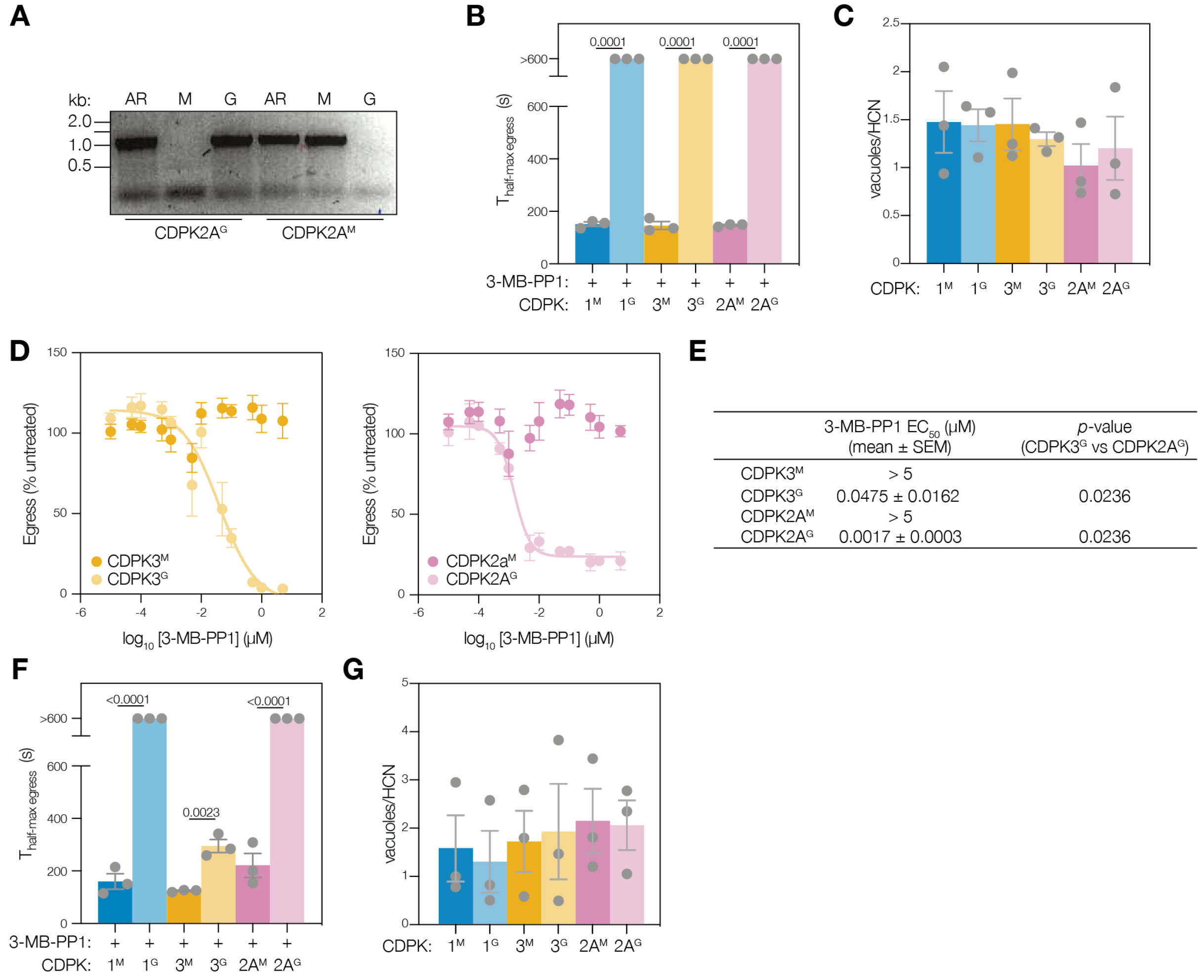

	3-MB-PP1 EC_{50} (µM) (mean ± SEM)	*p*-value (CDPK3^G vs CDPK2A^G)
CDPK3^M	> 5	
CDPK3^G	0.0475 ± 0.0162	0.0236
CDPK2A^M	> 5	
CDPK2A^G	0.0017 ± 0.0003	0.0236

Figure S1. Strain generation and extended egress assay results for chemical-genetic strategy. A. PCR of analog-sensitive kinase lines confirming allelic replacement (AR) and using allele-specific primers to distinguish Met (M) or Gly (G) gatekeeper residue. **B.** Time required for each strain to achieve half of the maximum A23187-stimulated egress when treated with 3-MB-PP1. Samples that did not achieve half-maximal egress within the observation window were plotted as >600 s. Bars represent the mean ± SEM for n = 3 biological replicates; significance calculated by unpaired t-test. **C.** Multiplicity of infection was determined counting vacuoles per host cell nucleus by immunofluorescence. Bars represent the mean ± SEM of n = 3 biological replicates; significance calculated by ANOVA. **D.** Dose-response of CDPK2A and CDPK3 AS-kinase lines to 3-MB-PP1, monitoring endpoint A23187-stimulated egress by LDH release. Graphs are egress of 3-MB-PP1–treated parasites as % of untreated. Points are the mean ± SEM for n = 3 biological replicates. **E.** EC_{50} (µM) for 3-MB-PP1 of CDPK2A and CDPK3 AS-kinase parasites; significance calculated by unpaired t-test across n = 3 biological replicates. **F.** Time required for each strain to achieve half of the maximum zaprinast-stimulated egress when treated with 3-MB-PP1. Samples that did not achieve half-max egress within the observation window were plotted as >600 s. Bars represent the mean ± SEM of n = 3 biological replicates; significance calculated by unpaired t-test. **G.** Multiplicity of infection was determined counting vacuoles per host cell nucleus by immunofluorescence. Bars represent the mean ± SEM for n = 3 biological replicates; significance calculated by ANOVA.

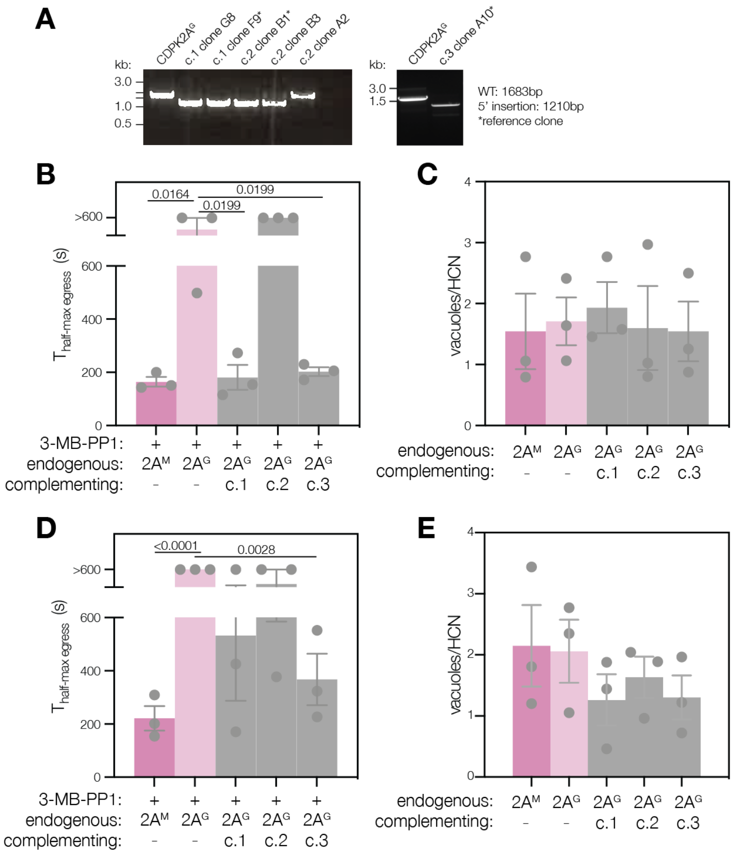

Figure S2. Trans-complementation of the CDPK2A^{G} allele. A. PCR of 5′ insertion junctions for complementing strains (c.1–c.3) for multiple recovered clones. Reference clone (*) indicates the clone used for all other experiments. **B.** Time required for each strain to achieve half of the maximum A23187-stimulated egress when treated with 3-MB-PP1. Samples that did not achieve half-max egress within the observation window were plotted as >600s. Bars represent the mean ± SEM for *n* = 3 biological replicates; significance calculated by unpaired *t*-test. **C.** Multiplicity of infection was determined counting vacuoles per host cell nucleus by immunofluorescence. Bars represent the mean ± SEM for *n* = 3 biological replicates; significance calculated by ANOVA. **D.** Time required for each strain to achieve half of the maximum zaprinast-stimulated egress when treated with 3-MB-PP1. Samples that did not achieve half-max egress within the observation window were plotted as >600s. Bars represent the mean ± SEM for *n* = 3 biological replicates; significance calculated by unpaired *t*-test. **E.** Multiplicity of infection was determined counting vacuoles per host cell nucleus by immunofluorescence. Bars represent mean ± SEM for *n* = 3 biological replicates; significance calculated by ANOVA.

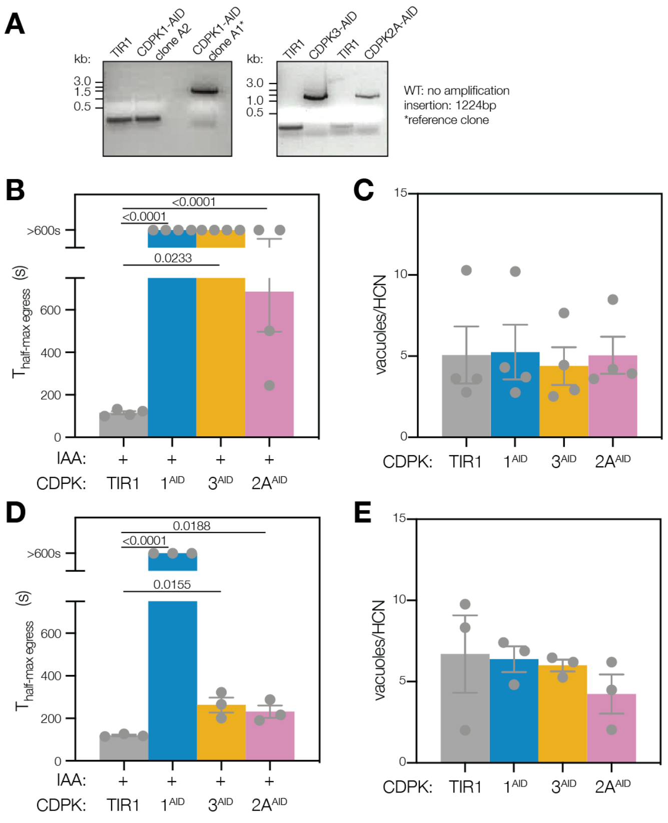

Figure S3. Strain generation and extended egress assay results for conditional-depletion strategy. A. PCR amplification of mNeonGreen-AID-Ty from tagged strains. Reference clone (*) indicates the clone used for all other experiments. **B.** Time required for each strain to achieve half of the maximum A23187-stimulated egress when treated with IAA. Samples that did not achieve half-max egress within the observation window were plotted as >600s. Bars represent mean ± SEM for *n* = 4 biological replicates; significance calculated by unpaired *t*-test. **C.** Multiplicity of infection was determined counting vacuoles per host cell nucleus by immunofluorescence. Bars represent mean ± SEM for *n* = 4 biological replicates; significance calculated by ANOVA. **D.** Time required for each strain to achieve half of the maximum zaprinast-stimulated egress when treated with IAA. Samples that did not achieve half-max egress within the observation window were plotted as >600s. Bars represent the mean ± SEM for *n* = 3 biological replicates; significance calculated by unpaired *t*-test. **E.** Multiplicity of infection was determined counting vacuoles per host cell nucleus by immunofluorescence. Bar represents mean ± SEM for *n* = 3 biological replicates; significance calculated by ANOVA.

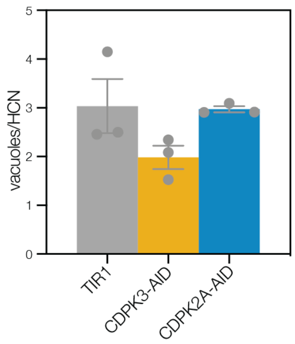

Figure S4. Multiplicity of infection for epistasis studies. Vacuoles per host cell nucleus determined by immunofluorescence. Bars represent mean ± SEM for *n* = 3 biological replicates; significance calculated by ANOVA.

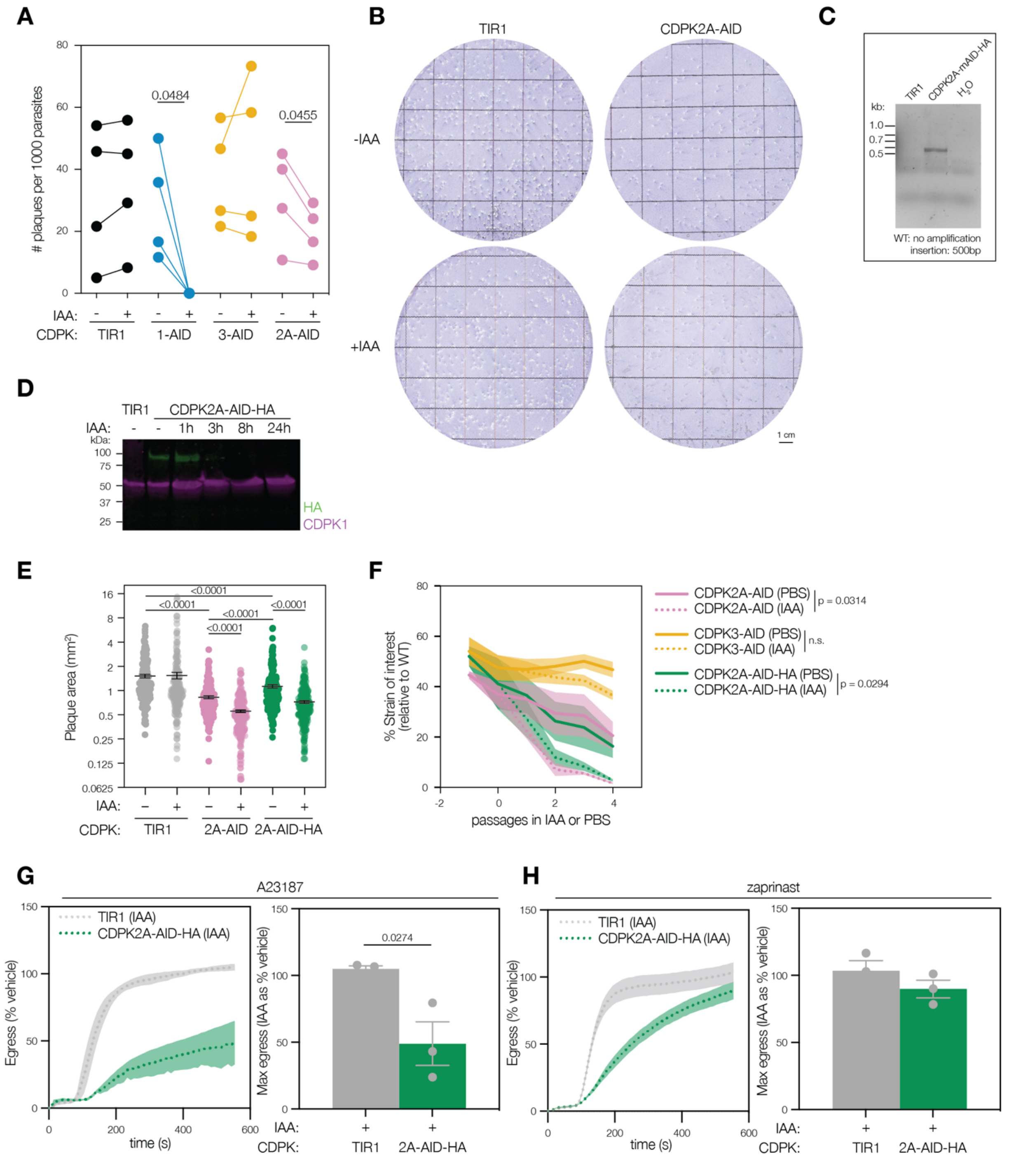

Figure S5. Extended effects of CDPK2A depletion across the lytic cycle. A. CDPK2A-depleted parasites form fewer plaques than the CDPK2A-AID tagged strain. Quantification of Fig. 5A. Points represent *n* = 4 biological replicates; significance calculated by paired *t*-test. **B.** CDPK2A-AID-tagged parasites form smaller plaques than the TIR1 parental strain, and plaque area is further decreased upon depletion of CDPK2A with IAA treatment. Scale bar is 1 cm. Quantified in Fig. 5B. **C.** PCR amplification of C-terminal AID-HA from CDPK2A-AID-HA

parasites. **D.** Immunoblot of TIR1 parental and CDPK2A-AID-HA parasite lines shows expression of HA epitope tag in CDPK2A-AID-HA line and depletion with IAA (1-24h, 500µM). CDPK1 is used as a loading control. **E.** CDPK2A-AID-HA tagged parasites form plaques that are intermediate between TIR1 parental and CDPK2A-AID. The plaque area defect is exacerbated by depletion of the kinase (+IAA). Scatter plot displays areas for 158-290 individual plaques per sample; mean ± SEM is overlaid for n = 3 biological replicates; significance calculated by unpaired one-tailed t-test. **F.** CDPK2A-AID and CDPK2A-AID-HA tagged parasites are outcompeted by wildtype (TIR1/IMC1-TdTom) parasites in 50:50 mixed cultures; parasites depleted of CDPK2A are outcompeted more than tagging alone. Line represents the mean ± SEM for n = 3 biological replicates; significance calculated by paired two-tailed t-test. **G.** CDPK2A-AID-HA parasites show diminished egress when stimulated with A23187. Kinetic traces are egress of IAA–treated parasites as % of vehicle. A23187 was added 1 s after the start of imaging. Line plots the mean ± SEM for n = 3 biological replicates. Maximum egress achieved by each strain during the observation window is displayed as % of vehicle. Bars represent the mean ± SEM of n = 3 biological replicates; significance calculated by unpaired t-test. **H.** CDPK2A-AID-HA parasites egress fully when stimulated with zaprinast. Kinetic traces are egress of IAA–treated parasites as % of vehicle. Zaprinast was added 1 s after the start of imaging. Line plots the mean ± SEM for n = 3 biological replicates. Maximum egress achieved by each strain during the observation window is displayed as % of vehicle. Bars represent the mean ± SEM of n = 3 biological replicates; significance calculated by unpaired t-test (N.S).

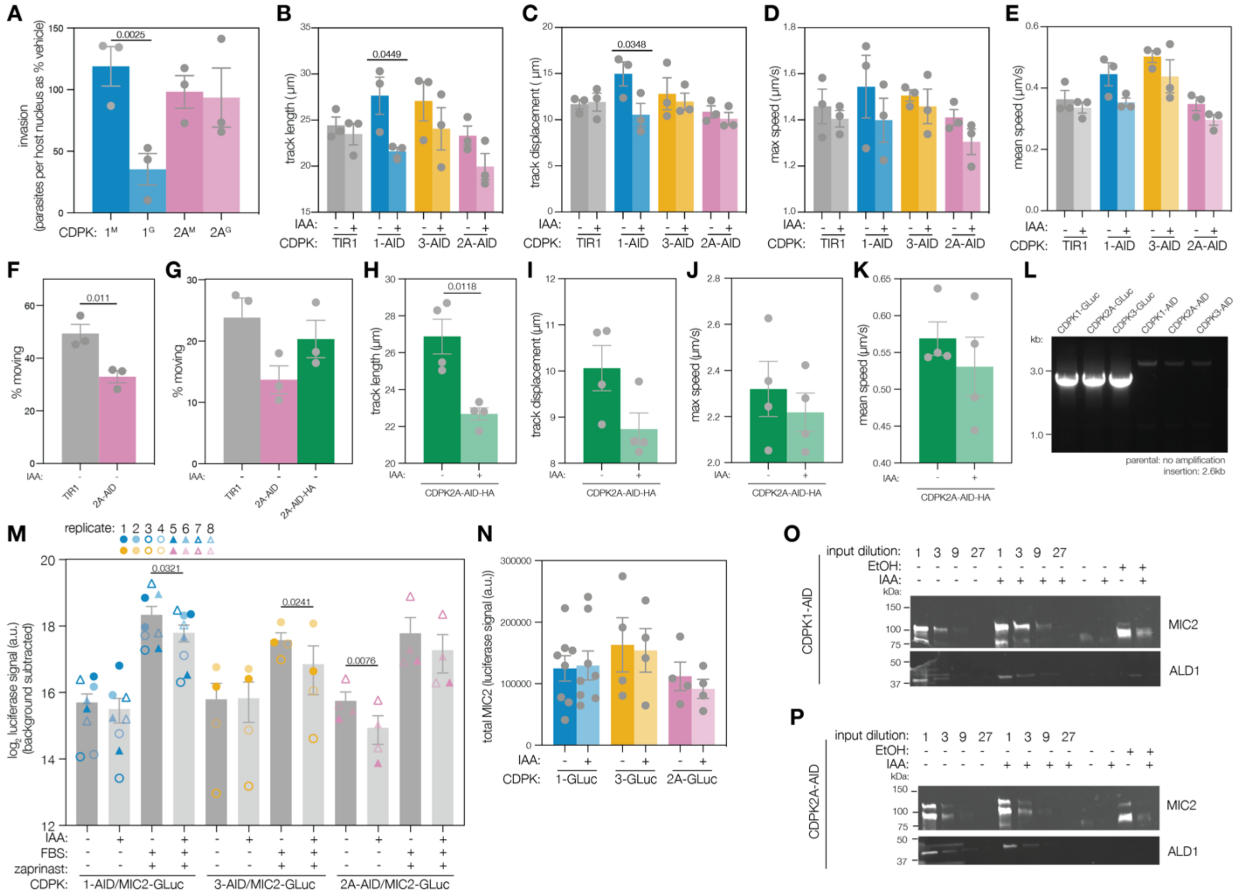

Figure S6. Extended effects of CDPK2A depletion or inhibition on motility and microneme discharge. A. CDPK2A inhibition with 3-MB-PP1 does not impact parasites' ability to invade host cells. Each point is the mean of 3 technical replicates; bars represent mean ± SEM for *n* = 3 biological replicates; significance calculated by paired *t*-test. **B.** Track length of moving parasites in 3D motility assays. Each point is the mean of 3 technical replicates; bars represent the mean ± SEM for *n* = 3 biological replicates; significance calculated by one-tailed unpaired *t*-test. **C.** Track displacement of moving parasites in 3D motility assays. Each point is the mean of 3 technical replicates; bars represent the mean ± SEM for *n* = 3 biological replicates; significance calculated by one-tailed unpaired *t*-test. **D.** Maximum speed of moving parasites in 3D motility assays. Each point is the mean of 3 technical replicates; bars represent the mean ± SEM of *n* = 3 biological replicates; significance calculated by one-tailed unpaired *t*-test. **E.** Mean speed of moving parasites in 3D motility assays. Each point is the mean of 3 technical replicates; bars represent the mean ± SEM of *n* = 3 biological replicates; significance calculated by one-tailed unpaired *t*-test. **F.** Comparison of the proportion of parasites gliding in 3D for TIR1 and CDPK2A-AID parasites without IAA. Each point is the mean of 3 technical replicates with 1011 and 1157 observations for TIR1 and CDPK2A-AID respectively; bars represent the mean ± SEM of *n* = 3 biological replicates; significance calculated by unpaired one-tailed *t*-test. **G.** Comparison of the proportion of parasites gliding in 3D for TIR1, CDPK2A-AID, and CDPK2A-AID-HA parasites without IAA. Each point is the mean of 3 technical replicates; bars represent the mean ± SEM of *n* = 3 biological replicates; significance calculated by ANOVA with Brown-Forsythe and Welch's corrections for multiple comparisons. **H.** Track length of moving parasites in 3D motility assays. Each point is the mean of technical

replicates; bars represent the mean ± SEM for *n* = 4 biological replicates; significance calculated by paired *t*-test. **I.** Track displacement of moving parasites in 3D motility assays. Each point is the mean of technical replicates; bars represent the mean ± SEM for *n* = 4 biological replicates; significance calculated by paired *t*-test. **J.** Maximum speed of moving parasites in 3D motility assays. Each point is the mean of technical replicates; bars represent the mean ± SEM of *n* = 4 biological replicates; significance calculated by paired *t*-test. **K.** Mean speed of moving parasites in 3D motility assays. Each point is the mean of technical replicates; bars represent the mean ± SEM of *n* = 4 biological replicates; significance calculated by paired *t*-test. **L.** PCR of 5′ insertion junction for GLuc strains. **M.** Depletion of CDPK1 or CDPK3decreases microneme content release following 5 minutes of stimulation with FBS and zaprinast. Gaussia luciferase activity was assayed in supernatants and background signal (from wells with no parasites) subtracted. Points represent individual replicates (*n* = 8 for CDPK1-AID, *n* = 4 for CDPK3-AID or CDPK2A-AID), with bars at mean ± SEM; significance calculated by paired 1-tailed *t*-test. **N.** Total MIC2 was the same across CDPK-GLuc strains with or without auxin. Gaussia luciferase activity was assayed in cell lysates generated from the same cell preparations used for microneme secretion assays. Points represent individual replicates (*n* = 8 for CDPK1-AID, *n* = 4 for CDPK3-AID or CDPK2A-AID), with bars at mean ±SEM; significance calculated by paired one-tailed *t*-test. **O.** Depletion of CDPK1 decreases secretion of MIC2 following 15 minutes of stimulation with FBS and ethanol. MIC2 in supernatants and paired lysate dilutions was assayed by immunoblot against MIC2; aldolase is used as a control for spontaneous lysis. **P.** Depletion of CDPK2A decreases secretion of MIC2 following 15 minutes of stimulation with FBS and ethanol. MIC2 in supernatants and paired lysate dilutions was assayed by immunoblot against MIC2; aldolase is used as a control for spontaneous lysis.
